# Maternal Preference, Mode of Delivery and Associated Factors among Women Who Gave Birth at Public and Private Hospitals in Hawassa City, Southern Ethiopia

**DOI:** 10.5334/aogh.2578

**Published:** 2019-08-19

**Authors:** Zelalem Tenaw, Zemenu Yohannes Kassa, Getinet Kassahun, Animut Ayenew

**Affiliations:** 1 Department of Midwifery, College of Medicine and Health Sciences, Hawassa University, ET; 2 McKing consulting corporation supporting Bill and Melinda Gates foundation, SS

## Abstract

**Introduction:**

Despite the advances in modern obstetrics care, maternal morbidity and mortality remains a big problem. Proper choice in the mode of delivery is necessary to tackle this problem. The aim of this study was to assess maternal preference, mode of delivery and associated factors among women who gave birth at public and private hospitals in Hawassa city, Southern Ethiopia, 2017.

**Methods:**

A hospital based cross sectional study was carried out from January 01–30/2017. A systematic sampling procedure was utilized, and 300 mothers who gave births were included in the study. Data entered to EPI data 3.5.1 and exported to version 20.0 software packages for social science analysis. The presence of association between independent and dependent variables was determined using odds ratio at 95% confidence interval by applying logistic regression model.

**Results:**

The prevalence of caesarean section was 49.3% (95% CI: 43.7–55.3). Mothers that have a monthly income above poverty line, having previous pregnancy complications, and current pregnancy problems have higher odds of using the caesarean section mode of delivery. Whereas utilization of partograph lower the odds of caesarean section mode of delivery. Having previous pregnancy complications had higher odds of maternal preference for caesarean section delivery whereas the utilization of partograph lowered the odds of maternal preference for Caesarean section delivery.

**Conclusion:**

The prevalence of caesarean section mode of delivery in Hawassa city was high compared with world health organization threshold. Monthly income above poverty line, previous pregnancy complications, Current obstetrics problems are increasing caesarean section delivery, whereas utilization of partograph is decreasing caesarean section delivery. Therefore, utilization of partograph could be lessening unnecessary caesarean section delivery.

## Introduction

The quality of obstetrics care is reflected by the magnitude of perinatal and maternal morbidity and mortality rates of a certain country, which is considered as one of the vital indicators of health status. Despite the advances in modern obstetrics care, maternal morbidity and mortality remains an international problem [1].

Worldwide, 3 million women give birth vaginally every year, many experiencing of problems like: perennial trauma from episiotomy, spontaneous obstetric lacerations, or both [2].

Sub-Saharan Africa has the highest maternal morbidity and mortality ratio (MMR), an average of 500 maternal deaths per 100,000 live births [3].

Ethiopian Federal Ministry of Health (EFMOH) has applied multi-pronged approaches to reduce maternal and newborn morbidity and mortality, improve access to and strengthen facility-based maternal and newborn services is one such approach, and is also a major issue of concern in Health Sector Transformation Plan 2015/16–2019/20 of Ethiopia [4].

Childbirth is a normal physiological process and a significant emotional event in a woman’s life. While proper choice of interventions is proven to be associated with the highest safety and effectiveness to reduce maternal and neonatal morbidity and mortality [5].

Caesarean section (C/S) is a surgical intervention designed to prevent or treat life threatening maternal or fetal complications [6]. A Caesarean section is often performed when a vaginal delivery would put the baby’s or mother’s life or health in danger. Some are also performed upon request without a medical reason to do [7].

Though the safety of caesarean section has improved, to date the morbidity rates are still high compared to the vaginal delivery [8].

According to world health organization (WHO) C/S rate in any population should lie within the range of 5–15%, and there is no justification in any specific geographic region to have more than 10–15% C/S births [9].

The C/S rate in Addis Ababa has increased considerably from 2.3% in 1995–1996 to 24.4% in 2009–2010. Since 2003 the rate persisted beyond the upper optimum level of WHO which is 15% [6].

Most of the studies concluded that vaginal delivery is safe and give a good selection for patients to have qualified assistance and careful management during delivery [10].

Vaginal delivery is the preservation and promotion of the normalcy of labor and delivery, including the woman’s active participation in the birth process [2].

Although fetal and maternal outcomes depends on the quality of care provided starting from the preconception period, the success of it is relied on timely and appropriately carried out intra partum care [11].

The appropriateness and ethical aspects of on-demand C/S has been hotly debated by obstetricians and women’s group for some years now. The debate has focused on the questions of risks and benefits of vaginal and C/S delivery and woman’s autonomy to choose her mode of delivery [6].

Most women expressed a preference for vaginal birth (70.8%). The majority of women (68.7%) made positive comments about vaginal birth, believing that it involved less suffering, better recovery, less risk, quicker, allow earlier discharge from the hospital, and better for women and their newborn babies [5]. Hence, evidence on maternal preference and mode of delivery among women is rare in Ethiopia. The aim of this study was to assess maternal preferences, mode of delivery and associated factors, in Hawassa city public and private hospitals, Southern Ethiopia.

## Methods and materials

A hospital based cross sectional study was carried out from January 01-30/2017 among women who gave birth at public and private hospitals in Hawassa city. Hawassa is the administrative city of Southern nation nationalities people regional state, which is located 275km away from Addis Ababa the capital city of Ethiopia. According to Hawassa city administration health department, the total population in 2016/2017 was expected to be 351,567 [12]. Out of the total population 170,510 (48.5%) were females. Women who were in child bearing age group (15–49) were 69,769; from this 12,167 were expected to be pregnant. Hospitals found in the city are: one governmental comprehensive specialized referral, one primary hospital and three private primary hospitals. The city had two governmental and one private hospital which give all delivery services (c/s and vaginal delivery).

The sample size was determined using the software Epi Info version 7 with the following assumptions: 95% confidence interval with 76.6% prevalence of vaginal deliveries [6], with (α = 0.05), 5% marginal error (d = 0.05). The final sample size was 304. Women who gave births at public and private hospitals in Hawassa city were included in the study. All public and private hospitals which gave vaginal and cesarean section delivery were included in the study. Sample size was proportionally allocated based on the number of births in the past one months. Using the expected 610 deliveries all hospitals in one month K value was 2. Systematic sampling procedure was used to interview study participants at postnatal ward. The first interviewee mother was selected by using simple random technique. Exit interview was conducted at a convenient and appropriate place.

The data was collected face to face using structured and pretested questionnaire interviews at the postnatal ward. The questionnaire was prepared by reviewing existing literatures, which consists of socio demographic characteristics, personal characteristics and obstetric history. Pretest was done 5% sample with similar sociodemographic characteristics of outset of study hospitals. Necessary amendment was made based on pretest findings accordingly.

Four (04) obstetric care providers who have BEmONC training were recruited and training was given for 02 days on the objective, relevance of the study, confidentiality of information, respondent rights, informed consent, and technique of interview; 01 health professional who have 1st degree (BSC midwife) were trained and supervise the data collection. Data entry was done using EPI Info 3.5.1 and exported to SPSS version 20.0 software for analysis. The presence of association between independent and dependent variables was determined using odds ratio with 95% confidence interval by applying logistic regression model.

## Results

### Socio-demographic characteristic and experiences of study participants

A total of 304 mothers participated in this study, with response rate of 98.7%. The ages of participants ranged from 18 to 45 years. The mean age (in years) of the study population was 27.02 ± 4.95 years. Sidama was a dominant ethnic group, which accounted 35.3% (n = 106). On the other hand, 45.7% (n = 137) of participants were housewives, whereas 31.3% (n = 94) of participants had graduated from college or university (Table 1).

**Table 1:** Socio demographic and economic characteristics of mothers who gave birth at public and private hospitals in Hawassa city hospitals, Southern Ethiopia 2017, (n = 300).

Variables		Frequency	Percentage

**Age**	18–22	68	22.7
	23–27	100	33.3
	28–32	99	33.0
	33–37	25	8.3
	38–45	8	2.7
**Religion**	Orthodox Christian	86	28.7
	Protestant	145	48.3
	Muslim	56	18.7
	Catholic	2	.7
	Jehovah witness	3	1.0
	other^Ω^	8	2.7
**Ethnicity**	Sidama	106	35.3
	Wolayta	45	15.0
	Amara	62	20.7
	Oromo	78	26.0
	Other^©^	9	3.0
**Marital status**	Single	9	3.0
	Divorced	3	1.0
	Widowed	1	.3
	Married	287	95.7
**Occupation of the mother**	House wife	137	45.7
	Government employed	73	24.3
	NGO employed	14	4.7
	Private	44	14.7
	Student	25	8.3
	Other^®^	7	2.3
**Occupation of spouse**	Farmer	55	18.3
	Government employee	115	38.3
	NGO employee	20	6.7
	Private	95	31.7
	Student	5	1.7
	Other^€^	10	3.3
**Residency**	Urban	233	77.7
	Rural	67	22.3
**Monthly income**	Extreme poor	60	20.0
	Under poverty	24	8.0
	Above poverty	216	72.0
**Educational status of mother**	Illiterate	48	16.0
	Read and write	7	2.3
	Primary school complete	66	22.0
	Secondary school complete	64	21.3
	Above secondary school	21	7.0
	Graduated from college or university	94	31.3

Other^Ω^: waqfetah, Traditional believer. Other^©^: Silte, kaffa. Other^®^: pension, merchant. other^€^: pension.

### Obstetric factor and experiences of study participants

About 88.3% (n = 265) of the participants were multi para, and 93.3% (n = 280) of pregnancy was planned. Concerning antenatal care 95% (n = 285) of the mother had ANC contact. Thirty percent of the mothers were referred due to ante partum hemorrhage, pregnancy induced hypertension, fetal distress and premature rupture of membrane (PROM) (Table 2).

**Table 2:** Obstetric characteristics of mothers who gave birth at public and private hospitals in Hawassa city hospitals, Southern Ethiopia 2017, (n = 300).

Variables		Frequency	Percentage (%)

Para	Nulipara	30	10.0
	Multipara	265	88.3
	Grandpara	5	1.7
Pregnancy	Planned	280	93.3
	Unplanned	20	6.7
Gestational age	Pre-term	26	8.7
	Term	267	89.0
	Post term	7	2.3
ANC follow up	Yes	285	95.0
	No	15	5.0
Number of ANC visit	No visit	15	5
	1	6	2
	2	34	11.3
	3	43	14.3
	4	150	50
	More than four	52	17.3
Referral status	Refer	90	30.0
	Not refer	210	70.0
Day of admission	Working day	233	77.7
	Other day	67	22.3
Time of admission	Morning	112	37.3
	Midday	69	23.0
	Evening	86	28.7
	Night	33	11.0
Previous pregnancy complication	Yes	72	24.0
	No	228	76.0
Types of previous pregnancy complication	c/s scar	36	12
	still birth/neonatal loss	27	9
	Over weight baby	4	1.3
	Other^◈^	5	1.7
Types of c/s	Elective c/s	42	14
	Emergency c/s	106	35.3
Maternal preference of mode of delivery	Caesarean section	38	12.7
	Spontaneous vaginal delivery	262	87.3

Other^◈^: Hypertension, obstructed labor.

### Mode of delivery

**Figure 1:**
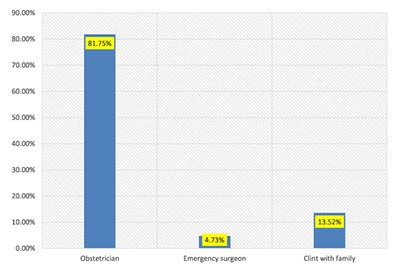
Decision for caesarean section delivery in Hawassa city hospitals.

The prevalence of caesarean section in Hawassa city was 49.3% (n = 148), from this 35.3% and 14% were emergency and elective caesarean section respectively. Meanwhile, 81.7% (n = 121) of the caesarean section delivery was decided by obstetricians (Figure 1).

### Maternal preferences for mode of delivery

Eighty-seven percent of the mothers preferred spontaneous vaginal delivery (Table 2).

### Factors associated with caesarean section delivery

Monthly income above poverty line, previous history of pregnancy complication, current obstetric problem, maternal preference of C/S delivery and Parthograph follow up were the factors associated with caesarean section delivery (Table 3).

**Table 3:** Factors associated with caesarean section delivery among mothers who gave birth at public and private hospitals in Hawassa city hospitals, Southern Ethiopia, 2017 (n = 300).

Characteristic/s		C/S delivery		OR (95% CI)		P-Value

		Yes	No	Crude	Adjusted	

Age	18–32	130	137	0.79 (0.38–1.63)		
	33–45	18	15	1.00		
Residency	Urban	118	115	1.27 (0.73–2.18)		
	Rural	30	37	1.00		
Marital status	Married	144	143	2.27 (0.68–7.52)		
	Not married	4	9	1.00		
Monthly Income	Above poverty	115	101	1.76 (1.05–2.94)*	3.78 (1.86–7.69)**	0.00
	Under poverty	33	51	1.00	1.00	
ANC follow-up	Yes	139	146	0.64 (0.22–1.83)		
	No	9	6	1.00		
Pregnancy	Planned	142	138	2.40 (0.89–6.43)		
	Un planned	6	14	1.00		
GA	Term	133	134	1.19 (0.58–2.46)		
	Pre/post-term	15	18	1.00		
Day of admission	Working day	110	123	0.68 (0.39–1.18)		
	Weekend	38	29	1.00		
Previous pregnancy complication	Yes	55	17	4.69 (2.56–8.59)*	4.63 (2.15–9.97)**	0.000
	No	93	135	1.00	1.00	
Condition of mother	Stable	141	147	0.68 (0.21–2.20)		
	Unstable	7	5	1.00		
Parthograph follow up	Yes	105	43	1.00	1.00	
	No	143	9	0.15 (0.72–0.33)*	0.12 (0.04–0.32)**	0.000
Amniotic fluid	Rupture	30	28	1.13 (0.64–1.99)		
	Intact	118	124	1.00		
Current obstetrics problem	Yes	50	26	2.48 (1.44–4.25)*	8.15 (4.25–15.62)**	0.000
	No	98	126	1.00	1.00	
Number of senior	Two	40	25	1.88 (1.07–3.23)*	0.57 (0.06–5.47)	0.63
	More than two	108	127	1.00	1.00	
Payment for delivery	Yes	39	20	2.36 (1.30–4.29)*	4.55 (0.44–47.34)	0.20
	No	109	132	1.00	1.00	
Parity	Prime Para	18	12	1.62 (0.75–3.48)		
	Multi Para	130	140	1.00		
Types of hospital	Public	109	131	0.45 (0.25–0.80)*	0.56 (0.28–1.07)	
	Private	39	21	1.00		

* P-value ≤ 0.25. ** Adjusted for socio-demographic characteristics and some concepts of Mode of delivery.

### Factors associated with maternal preference for caesarean section delivery in Hawassa city

Previous pregnancy complication and having no Parthograph follow-up were the factors associated with maternal preference for Caesarean section delivery (Table 4).

**Table 4:** Factors associated with maternal preference for caesarean section delivery among who gave birth at public and private hospitals in Hawassa city hospitals, Southern Ethiopia 2017, (n = 300).

Characteristics		Maternal Preference C/S delivery		OR (95% CI)		P-Value

		Yes	No	Crude	Adjusted	

Age	18–32	35	232	1.51 (0.19–2.29)		
	33–45	3	30	1.00		
Residency	Urban	35	198	0.27 (0.08–0.89)*	2.45 (0.61–10.12)	0.204
	Rural	3	64	1.00	1.00	
Marital status	Married	36	251	0.79 (0.17–3.70)		
	Not married	2	11	1.00		
Monthly Income	Above poverty	33	183	2.85 (1.07–7.57)*	2.24 (0.69–7.26)	0.178
	Under poverty	5	79	1.00	1.00	
ANC follow-up	Yes	36	249	0.94 (0.20–4.34)		
	No	2	13	1.00		
Pregnancy	Planned	36	244	1.33 (0.29–5.96)		
	Un planned	2	18	1.00		
GA	Term	36	231	2.42 (0.55–10.50)	1.99 (0.37–10.72)	0.42
	Pre/post-term	2	31	1.00	1.00	
Previous Pregnancy complication	Yes	25	47	8.80 (4.19–18.45)*	10.02 (4.50–22.33)**	0.000
	No	13	215	1.00	1.00	
Parthograph follow up	Yes	23	225	0.25 (0.12–0.53)*	0.25 (0.10–0.62)**	0.002
	No	15	37	1.00	1.00	
Amniotic fluid	Rupture	4	54	0.45 (0.15–1.33)		
	Intact	34	208	1.00		
Current obstetrics problem	Yes	11	128	0.43 (0.20–0.89)*	0.53 (0.22–1.29)	0.164
	No	27	134	1.00	1.00	
Payment for delivery	Yes	9	50	1.32 (0.59–2.96)		
	No	29	212	1.00		
Parity	Prime Para	2	28	0.46 (0.17–2.03)		
	Multi Para	36	234	1.00		

* P-value ≤ 0.25. ** Adjusted for socio demographic characteristic/s and some concepts of maternal.

## Discussion

Cesarean section is a reproductive concern both in developed and developing countries. It is increasing through time in many countries without health gain. Evidence suggest that increment of Caesarean section has not decreased maternal and neonatal morbidity and mortality. The purpose of this study was to assess maternal preference, mode of delivery and associated factors at public and private hospitals in Hawassa city Southern Ethiopia. The prevalence of caesarean section in Hawassa city is 49.3% (n = 148), from this 35.3% were emergency caesarean section. This finding is inconsistent to the WHO recommendation and the study done in Addis Ababa 24.4% [6].

The possible explanation might be most of the Hawassa city public and private hospitals used as referral from Oromia and south regions rural areas and the women may come with obstetrical complications.

The prevalence of Caesarean section delivery in private hospital (65%) was higher than public hospitals (45%). The difference might be private hospital users are economically good and can afford the payment. They might be choose the Caesarean section to escape from labor pain.

In Ethiopia obstetrics related cares at public health institutions is freely available, while in private hospitals the average charge for spontaneous vaginal delivery was 34 USD and for caesarean section was 130 USD.

Eighty-seven (n = 261) of the mothers were preferred vaginal delivery. This finding is higher than the study conducted in Brazil where 70.8% of mothers preferred vaginal delivery [5].

Maternal preference for Caesarean section delivery increases significantly with previous history of pregnancy complication (AOR = 10.02, 95% CI [4.50–22.33]) this might be due to a bad experience from a previous pregnancy complication. Women who had a partograph follow up was less likely to have a caesarean section delivery (AOR = 0.25, 95% CI [0.10–0.62]). The possible reason might be during the Partograph follow-up unnecessary decision for caesarean section would be avoided due to strict follow-up.

Despite 87% (n = 261) of the mothers who preferred spontaneous vaginal delivery, 13.52% (n = 41) of them decided their mode of delivery. Obstetricians decided 81.75% (n = 245) the Caesarean section mode of delivery. This might be due to work overload and payment-related issues. One obstetrician had work both in private and public hospitals; due to this, there is no time to follow up. This could lead to the increase of using the caesarean section. Having monthly income above poverty line 3.78 times higher odds of Cesarean section, having previous pregnancy complication 4.63 times higher odds of Cesarean section, having current obstetrics problem 8.15 times higher odds of Cesarean section. However, having a Partograph follow up shows a 0.25 times lower chance of Caesarean section. In this finding, monthly income coincided with the study done in Eastern Ethiopia [13].

The strength of this study is adding a variable like Partograph follow ups and maternal preference of delivery. The limitation of this study is not involving obstetric health care providers in the study, self-reporting data which means non-observation.

## Conclusion

The prevalence of the Caesarean section mode of delivery in Hawassa city was high compared with the World Health Organization threshold. Monthly income above the poverty line, previous pregnancy complications and current obstetrics problems are increasing Caesarean section delivery, whereas utilization of Partograph is decreasing Caesarean section delivery. Therefore, utilization of Partograph could lessen unnecessary caesarean section deliveries.

## Data Accessibility Statement

We sent all which is available, as there is no remaining data and materials.

## Abbreviations

AOR – Adjusted odds ratio

BEmONC – Basic emergency obstetrics and newborn care

C/S – Caesarean section

EBR – Ethiopian Birr

E.C. – Ethiopian calendar

EFMOH – Ethiopian Federal Ministry of Health

MMR – Maternal mortality ratio

SPSS – Statically package of social science

SNNPRS – Southern nation nationalities people regional state

WHO – World Health Organization
